# Chocolate-Induced Sinus Tachycardia: A Sweet Surprise

**DOI:** 10.7759/cureus.98908

**Published:** 2025-12-10

**Authors:** Meena Rabi, Luna Khan, Adil Rabi

**Affiliations:** 1 Cardiology, Royal Bolton Hospital, Bolton, GBR; 2 Emergency Department, Lancaster University, Blackpool, GBR; 3 Emergency Medicine, Blackpool Victoria Hospital, Blackpool, GBR

**Keywords:** caffeine, cardiac arrythmia, chocolate, dietary factors, methylxanthines, palpitations, sinus tachycardia, theobromine

## Abstract

Sinus tachycardia is a common clinical presentation caused by various physiological or pathological factors and triggers. Determining the underlying cause of this presentation is crucial for providing effective and appropriate management.

We report the case of a 63-year-old male who presented to the emergency department with palpitations and dizziness. He had no associated symptoms such as chest pain, syncope, or breathlessness. Electrocardiography showed sinus tachycardia without any additional abnormalities. Laboratory investigations, including inflammatory markers, thyroid function tests, and cardiac enzymes such as d-dimer and troponin, were within normal limits. Additionally, a detailed history revealed excessive chocolate consumption of approximately 180 grams nightly for two weeks. He was treated with bisoprolol, intravenous fluids, and dietary counselling, which led to the resolution of his symptoms.

Chocolate contains methylxanthines, such as caffeine and related stimulants, which can increase heart rate and blood pressure, and may induce arrhythmias by stimulating the sympathetic nervous system. These effects can occur even in individuals without pre-existing cardiovascular disease. Consequently, dietary factors should not be overlooked during the evaluation of unexplained tachycardia, as they may play a significant role in its aetiology.

This case highlights the importance of considering dietary factors, like chocolate and caffeine intake, when assessing patients with tachycardia. Identifying reversible, lifestyle-related causes can prevent unnecessary investigations and ensure the initiation of effective management.

## Introduction

Sinus tachycardia is a common presentation and electrocardiographic finding. It is defined as a regular heart rhythm and rate faster than 100 beats per minute with normal P-wave morphology and axis [1]. This can occur in response to various factors, including anxiety, exercise, infection, endocrine abnormalities, or the use of certain medications. The heart rate increases when the sinoatrial node accelerates electrical signals to the atrioventricular node. This then transmits these impulses through the bundle of His to the ventricles of the heart [2]. The list of differential diagnoses is extensive, and prompt identification of the underlying cause is essential for timely investigation and management.

Caffeine and theobromine, found in chocolate, energy drinks, and other caffeinated drinks such as coffee and tea, are stimulants known to increase heart rate and cardiac output [3,4]. This case describes isolated sinus tachycardia associated with excessive chocolate consumption in an otherwise healthy adult, highlighting the importance of a thorough dietary history when investigating unexplained tachycardia.

## Case presentation

We present the case of a 63-year-old man with no prior cardiac history who presented to the Emergency Department at 10:00 a.m. with palpitations that began at 01:00 a.m. He reported dizziness and paraesthesia in his fingers but denied shortness of breath, chest or abdominal pain, headache, or visual changes. His past medical history included a diagnosis of human immunodeficiency virus (HIV) infection and obstructive sleep apnoea. He was receiving antiretroviral therapy with Odefsey (emtricitabine/rilpivirine/tenofovir alafenamide) and demonstrated good adherence. He is a non-smoker and denies recreational drug use or alcohol consumption. Further history revealed nightly consumption of 200 grams of dark chocolate for the preceding two weeks, totalling 2.8 kilograms.

Initial observations included the following: heart rate of 152 beats per minute, blood pressure of 159/106 mmHg, a respiratory rate of 17 breaths per minute, and oxygen saturation (SpO₂) of 99% on room air. Physical examination was unremarkable, with no evidence of infection, dehydration, or cardiac pathology such as heart failure.

Laboratory investigations showed normal cardiac enzymes and inflammatory markers. All other blood results, including liver, renal and thyroid function tests, remained normal. Complete laboratory results are summarised in Table 1, including corresponding reference ranges. No abnormalities were identified. 

**Table 1:** Summary of laboratory investigations performed on admission. All parameters were within normal limits.

Parameter	Result	Reference Range	Interpretation
D-dimer	279	<500 ng/mL	Normal
Troponin	4.9	<11 ng/L	Normal
C-reactive protein (CRP)	2.6	0-5 mg/L	Normal
White blood cell count (WBC)	7.1	4.0-11.0 x 10⁹/L	Normal

**Figure 1:**
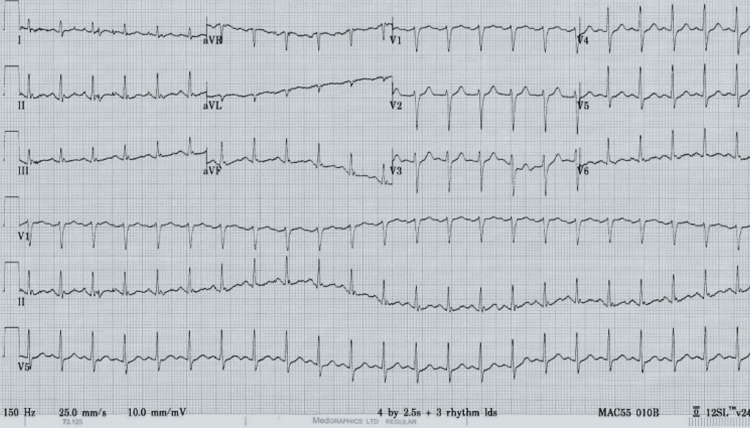
Resting 12-lead ECG showing sinus tachycardia

The resting 12-lead ECG displayed sinus tachycardia with no additional features (Figure 1).

The patient received intravenous fluids and a single dose of bisoprolol fumarate, a beta blocker, to alleviate palpitations. He was counselled regarding the probable association between excessive chocolate intake and his symptoms, and advised to reduce consumption. No structural cardiac abnormalities were found, and the patient was treated conservatively. Upon stabilisation and normalisation of his heart rate, he was discharged, and a follow-up appointment was arranged with his General Practitioner (GP).

## Discussion

Sinus tachycardia is a physiological response to increased sympathetic activity or reduced parasympathetic tone. It typically reflects an underlying metabolic or systemic process such as infection, endocrine disorders, or anaemia, rather than primary cardiac pathology. Chocolate contains methylxanthines, particularly caffeine and theobromine, which act as antagonists of adenosine receptors. This reduces adenosine-mediated vasodilation and bradycardia, thereby increasing heart rate and blood pressure [3,4]. These compounds also stimulate the sympathetic nervous system by increasing circulating cortisol and catecholamines, particularly adrenaline and noradrenaline. This stimulation can precipitate palpitations or tachyarrythmias, especially when consumed in excess [3,5].

Despite the well-documented link between caffeine and arrhythmias, doctors often overlook dietary influences that can affect heart rate, such as caffeine, alcohol intake, and chocolate ingestion, when considering factors. A previous case of chocolate-induced supraventricular tachycardia highlights the clinical relevance of investigating such potential dietary triggers [6]. Additionally, there have been reports describing more serious arrhythmias linked to chocolate consumption, such as atrial fibrillation triggered by a combination of high chocolate intake with β-agonist overuse. This demonstrates that methylxanthines can lead to a range of cardiac rhythm disturbances [7]. 

Additional research on stimulants provides supporting evidence: metabolic studies demonstrate that cocoa-derived methylxanthines influence autonomic tone and cardiovascular responses [8]. Chemical analyses confirm that many dark chocolate products contain amounts of theobromine and caffeine capable of producing physiological effects in susceptible individuals [9].

In this case, the patient developed isolated sinus tachycardia without progression to more complex arrhythmias or showing evidence of myocardial injury. Common causes of sinus tachycardia, including pulmonary embolism, myocardial infarction, and infection, were ruled out in this patient based on laboratory and ECG findings. The clear temporal link between excessive chocolate consumption and symptom resolution after cessation strongly supports a methylxanthine-mediated mechanism. Importantly, while caffeine often receives more clinical attention, dark chocolate can contain sufficient theobromine to provoke a similar or even more pronounced stimulatory effect [3,5]. This distinction is clinically relevant, as patients may not recognise their chocolate intake as a source of stimulant exposure. 

This case reinforces the importance of obtaining a detailed dietary and lifestyle history when evaluating unexplained tachycardia, particularly when initial laboratory investigations are normal. Identifying modifiable triggers such as chocolate or caffeine intake can prevent unnecessary testing, reduce patient anxiety, and enable simple, effective management strategies through dietary modification alone, thereby improving patient care.

## Conclusions

This case demonstrates that excessive chocolate consumption can provoke sinus tachycardia through methylxanthine-induced sympathetic stimulation, even in people with no underlying cardiac disease. Recognising dietary stimulants as potential contributors to tachycardia is essential, particularly when initial investigations are unremarkable. A comprehensive lifestyle history, including assessment of chocolate, caffeine, and other stimulant intake, can help identify reversible causes, avoid unnecessary diagnostic testing, and guide appropriate management and care. Clinicians should maintain a high index of suspicion for dietary factors in cases of otherwise unexplained tachycardia, as simple modifications can lead to rapid symptom resolution and improved patient outcomes.
